# Tunable Infrared Detection, Radiative Cooling and Infrared-Laser Compatible Camouflage Based on a Multifunctional Nanostructure with Phase-Change Material

**DOI:** 10.3390/nano12132261

**Published:** 2022-06-30

**Authors:** Mingyu Luo, Xin Li, Zhaojian Zhang, Hansi Ma, Te Du, Xinpeng Jiang, Zhenrong Zhang, Junbo Yang

**Affiliations:** 1 Guangxi Key Laboratory of Multimedia Communications and Network Technology, School of Computer, Electronics and Information, Guangxi University, Nanning 530004, China; luomingyu20@163.com; 2 Center of Material Science, College of Liberal Arts and Sciences, National University of Defense Technology, Changsha 410073, China; lixin1010106@163.com (X.L.); zhangzhaojian@nudt.edu.cn (Z.Z.); mahansi21@nudt.edu.cn (H.M.); dute95@foxmail.com (T.D.); jackson97666@163.com (X.J.)

**Keywords:** multifunctional, infrared detection, thermal management, compatible camouflage, nanomaterials

## Abstract

The nanostructure composed of nanomaterials and subwavelength units offers flexible design freedom and outstanding advantages over conventional devices. In this paper, a multifunctional nanostructure with phase-change material (PCM) is proposed to achieve tunable infrared detection, radiation cooling and infrared (IR)-laser compatible camouflage. The structure is very simple and is modified from the classic metal–dielectric–metal (MIM) multilayer film structure. We innovatively composed the top layer of metals with slits, and introduced a non-volatile PCM Ge_2_Sb_2_Te_5_ (GST) for selective absorption/radiation regulation. According to the simulation results, wide-angle and polarization-insensitive dual-band infrared detection is realized in the four-layer structure. The transformation from infrared detection to infrared stealth is realized in the five-layer structure, and laser stealth is realized in the atmospheric window by electromagnetic absorption. Moreover, better radiation cooling is realized in the non-atmospheric window. The proposed device can achieve more than a 50% laser absorption rate at 10.6 μm while ensuring an average infrared emissivity below 20%. Compared with previous works, our proposed multifunctional nanostructures can realize multiple applications with a compact structure only by changing the temperature. Such ultra-thin, integratable and multifunctional nanostructures have great application prospects extending to various fields such as electromagnetic shielding, optical communication and sensing.

## 1. Introduction

As we all know, thermal radiation is a natural phenomenon everywhere in our life. It propagates in the form of electromagnetic (EM) waves and undertakes the important function of heat transfer at the same time, among which the infrared (IR) band has the most significant thermal effect. Since most of the infrared radiation of objects in nature is incoherent light, their radiation is unpolarized in the whole wide spectrum, and does not change with angle and random direction. The control of thermal radiation can only be achieved by changing the temperature of the object, which has great limitations in practical applications. In fact, the ability to control EM absorption and thermal radiation is important in many applications, including infrared detection [1,2], radiative cooling [3,4,5], solar steam engines [6,7], spectral sensors [8], thermal management [9,10] and thermal camouflage [11,12,13,14,15,16,17].

In recent decades, with the rapid development of nanotechnology, many interesting phenomena of manipulating EM waves have been generated. Through the interaction between subwavelength size structures and light by using metasurfaces (MSs), the free regulation of the infrared thermal radiation of objects has become a reality [18,19,20,21]. More and more attention has been paid to the law of light–matter interaction at the micro/nano scale and its application in light modulation and detection [22,23,24]. Therefore, by combining the modulation effect of photonic devices with the infrared photothermal effect, the design of effective micro/nano structures is conducive to manipulating the optical form of the thermal radiation of objects in the fields of infrared stealth [25], nano manufacturing [26] and solar energy utilization [27]. For example, based on the principles of metamaterials (MMs) [28,29,30], PCMs and plasmonics [31], thermal radiation is enhanced or suppressed at specific wavelengths. These photon structures show strong absorption dependence based on the resonant wavelength of electromagnetic waves. By reasonably designing the shape and size of micro-nano structures, we can independently choose the intensity, wavelength and bandwidth of radiation according to Kirchhoff’s law of thermal radiation, so as to obtain the adjusted thermal radiation spectrum. At present, with these characteristics, the response wavelength of micro-nano structures can be extended to various bands of visible light [32,33], infrared [34,35], terahertz [36,37], millimeter wave [38,39,40] and microwave [41,42,43]. Based on this, micro-nano devices are widely used in optical computing [44], night infrared imaging [45], infrared camouflage [46,47], optical communication [48] and other applications. According to Wien’s displacement law of thermodynamics,

(1) $$\lambda_{max}\cdot T=2898\mu m\cdot K,$$

For the black body, the peak wavelength of thermal radiation is in the infrared band (wavelength range: 700 nm–1 mm) under normal conditions, especially in the mid-infrared band (wavelength range: 1.4–14 μm). Therefore, the reconnaissance, detection and imaging technology in the mid-infrared band began to develop rapidly, and has been widely applied in various fields, such as infrared photoelectric sensors, infrared detectors and infrared thermal imagers [49]. In addition, the applications for military reconnaissance and stealth related to the middle infrared have also made this band become the focus of competing research.

Due to the physical effects, such as surface plasmon resonance (SPR) and Fabry Perot (FP) cavity resonance, the intrinsic emissivity of the object can be changed by changing the material, structure and size of the micro-nano photonic device. Thus, adjustable infrared absorption and thermal radiation can be realized, and even compatible camouflage of two different bands can be integrated in the same device. For example, Du Kaikai et al. have proposed a selective microbolometer based on metamaterial absorbers (MAs); MAs provide a new method for heat distribution adjustment and monitoring of microbolometers, and show great promise in photothermal imaging systems. Therefore, micro-nano structures make great contributions to infrared detection in the specific wavelength region of mid-far infrared [50]. Zhu Huanzheng et al. realized multispectral camouflage, wavelength selective emission and microwave absorption by using micro-nano structures such as multilayer films (including ZnS/Ge) and metasurfaces (including Cu-ITO-Cu), providing an idea for multifunctional compatible stealth [51]. It is important to note, however, that the selectivity of specific wavelengths in these studies all depends on the shape, size and period of the corresponding micro-nano structure. That is to say, once these micro-nano structures are manufactured, their corresponding resonant wavelength and absorption spectrum are determined at the same time and cannot be changed. Therefore, how to realize an adjustable multifunctional application on the basis of a fixed structure is very important for dynamic compatibility stealth. PCMs have a good prospect in active dynamic tuning, such as Ge_2_Sb_2_Te_5_ (GST), VO_2_, Ge_2_Se_2_Sb_4_Te_1_ (GSST) and Sb_2_S_3_, which have two states (crystalline and amorphous) with physical properties that are obviously different. In particular, the dielectric constant of GST varies greatly before and after phase transformation, and GST has been widely used in a variety of photoelectric devices, such as absorbers, chiral metamaterials, optical switches [52,53], tunable optical metasurfaces [54] and multifunctional devices [55]. Based on the above requirements, we consider combining PCMs and MSs to achieve complex multifunctional functions on a very simple micro/nano structure, while controlling the absorption/radiation intensity only by temperature changes without changing the structure and size, so as to achieve different practical applications.

In this work, we design and study a multifunctional metal–dielectric–metal (MIM) sandwich structure. Firstly, the conventional MIM multilayer film structure is composed of Au/Al_2_O_3_/Au on the bottom silicon substrate, and the top layer of the structure is innovatively composed of metals with periodic slits. It can be used to stimulate nanogap plasmon resonance and surface plasmon resonance in combination with a dielectric spacer to realize an infrared detection function. Subsequently, the PCM GST is added to the multilayer structure to form the Au/Al_2_O_3_/GST/Au/Si structure. By controlling the temperature, the GST changes dynamically and continuously between amorphous, intermediate and crystalline states, and changes the absorptivity/emissivity of the device to the IR-laser band, so as to achieve different functions. Compared with previous work, our design overcomes the disadvantages of being untunable, single function and difficult to integrate. It can realize many functions such as tunable infrared detection, radiation cooling and infrared-laser compatible camouflage under a simple structure with active control. This will greatly contribute to the development of optical communication, radiation cooling, adjustable electromagnetic wave control and various thermal camouflage technologies for military purposes.

## 2. Materials and Methods

**Figure 1:**
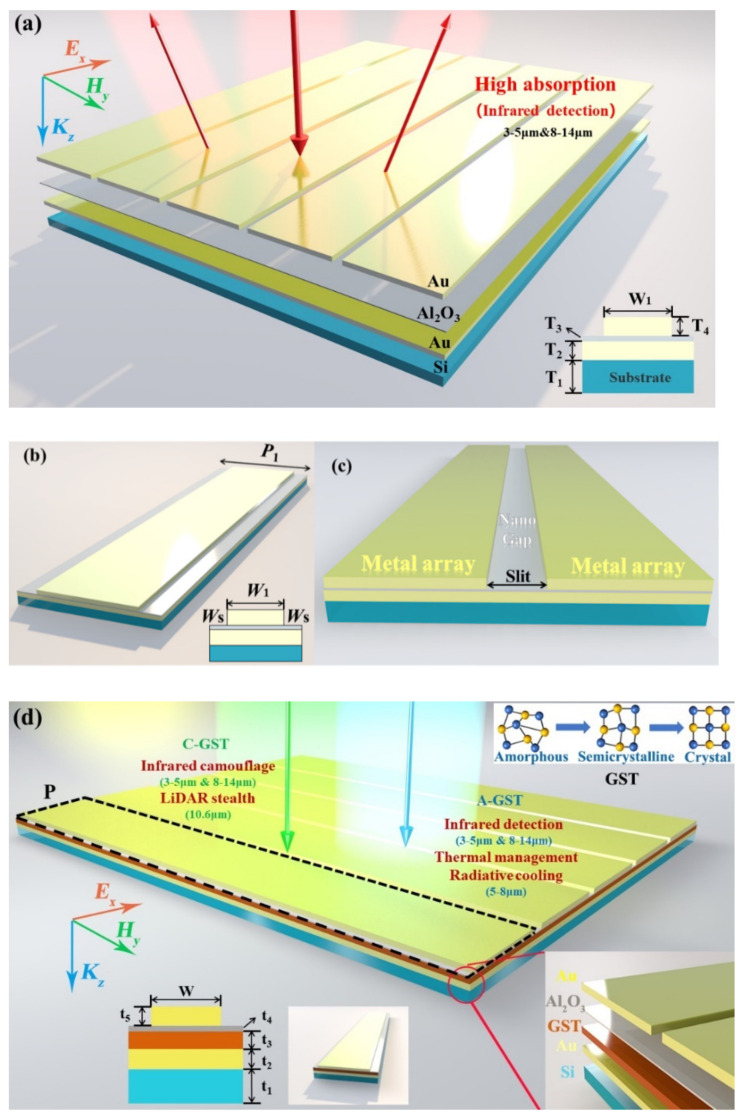
Schematic diagram of the geometric model of the designed multifunctional device: (**a**) three-dimensional view of the periodic four-layer structure, (**b**) view of the four-layer unit structure (with the metal array as the center of symmetry) and its dimensions, (**c**) view of the five-layer unit structure (with the slit as the center of symmetry) and (**d**) 3D view of the periodic five-layer structure.

Figure 1 shows the unit cell and periodic structure of the designed multifunctional device, respectively. By changing the number of layers, complex multifunctional applications can be realized. First, the proposed periodic device with dual-band infrared detection consists of four layers. Using magnetron sputtering and physical deposition, multilayer films can be obtained; the description of the fabrication steps is detailed in Supplementary Materials Information S2. Silicon substrate and dielectric layer alumina films are obtained by radio frequency sputtering (RF sputtering), gold films are obtained by direct current sputtering (DC sputtering) and GST thin films are obtained by the three-target co-sputtering method. The GST films obtained by this method are in the amorphous state at room temperature. After annealing at 160 °C (433.15 K), the nuclei inside the GST will join together to form a regular crystal structure. Thus, the amorphous GST (aGST) will transform into a crystalline GST (cGST) [56]. If the temperature continues to rise, the regular crystals inside the cGST slowly turn into disordered nuclei again, and the cGST returns to aGST after undergoing rapid annealing at 640 °C (913.15 K) [57]. The whole reversible phase change process is continuously related to the heating temperature and heating time, and this process and the temperature profile for the reversible phase change operation can be described in various experimental ways [58,59,60,61]. For example, the electrical pulsing schemes and meta-atom temperature profiles are shown by designing an electrothermal metasurface switching [58], and the corresponding temperature responses can be obtained when the GST is heated by a resistive microheater integrated with a phase-change metasurface [59]. Since GST is non-volatile, once the phase transition is completed, it can maintain the corresponding state at room temperature for a long time, so our research was carried out at room temperature. Meanwhile, since Si, Au and Al_2_O_3_ all have high melting and boiling points, the annealing process of GST does not affect the final research results [62]. If we do not change the temperature directly, the phase change can also be stimulated by other methods, such as electrical tuning [60], trains of femtosecond pulses [63] and laser pulse control [64]. As shown in Figure 1a, from bottom to top are Si, Au, Al_2_O_3_ and Au, respectively. The top layer is alternately composed of gold arrays with slits, combined with the dielectric layer Al_2_O_3_ in the middle to generate nanogap resonance and plasmon resonance to achieve high absorption at the corresponding wavelength. In order to simulate the absorption spectrum of the nanogap resonance, we studied the electromagnetic response of the structure, and obtained the optimal parameters after modeling and optimization of the structure through the simulation and numerical calculation of the physical field with commercial software (Lumerical, FDTD Solutions). As shown in Figure 1b, the width of the single gold array at the top of the four-layer unit structure is *W*_1_ = 2 μm, the width of the half slits on the left and right sides are *W*_S_ = 50 nm, and the single period of the top periodic structure is *P*_1_ = 2.1 μm. The thickness of the multilayer film structure from bottom to top is: *T*_1_ = 100 nm, *T*_2_ = 50 nm, *T*_3_ = 20 nm, and *T*_4_ = 50 nm. Next, in Figure 1c,d, we can observe that after adding the GST layer, the thicknesses of the layers from bottom to top are: *t*_1_ = 100 nm, *t*_2_ = 50 nm, *t*_3_ = 50 nm, *t*_4_ = 10 nm, and *t*_5_ = 50 nm. For the single gold array on top of the five-layer unit structure, the width *W* = 2.7 μm and the period *P* = 2.8 μm. Figure 1d shows that with the addition of the GST layer, the designed structure can achieve different functions in different states and different bands, and complete multifunctional applications.

To evaluate the electromagnetic response of the designed multifunctional device, we performed an analysis using the finite-difference time-domain (FDTD) method. We used a normal incident plane wave (3–14 µm) as the light source to excite the structure in the mid-infrared band. A flux monitor (frequency domain power monitor) was placed above the light source to measure reflectance. At the same time, a flux monitor was set up in the X–Z plane to monitor the distribution of electric and magnetic fields. In the simulation process, we took a unit structure of the periodic overall structure as the calculation object. Periodic boundary conditions were chosen in the X and Y directions, while the boundary conditions in the Z direction were set to perfectly matched layers, using a perfect electrical conductor at the bottommost layer of the structure. Planar light waves generated by a light source were incident on the structural model, and the incident electromagnetic waves entered the subwavelength volume space at resonant wavelengths under the action of the top metal array. The interstitial plasmonic mode was excited in the alumina gap at infrared wavelengths, and then the reflected light waves were received in the monitor. The calculation equation of the absorption efficiency *A(λ)* of the multifunctional device can be written as

(2) Aλ=1−Rλ−Tλ, 

where *R(λ)* and *T(λ)* are the reflectance and transmittance, respectively. It can be seen from Equation (2) that if the absorption rate is to be increased, the reflectance and transmittance need to be decreased. In our electromagnetic simulation, the substrate of the MIM structure in the model was made of a gold film as a metal mirror to effectively reflect light, so the transmittance of the entire structure was close to zero. Combined with Equation (2), the absorption rate of the multifunctional device was: *A(λ)* = 1 − *R(λ)*. The dispersion curves and dielectric constants of the materials used in the multifunctional device unit structure come from related work. The dielectric constants of Al_2_O_3_, Si and Au at mid-infrared wavelengths (3–14 μm) were fitted by reference [65]. The extinction coefficient and refractive index were obtained from the study by J. Tian et al. [61].

## 3. Results and Discussion

**Figure 2:**
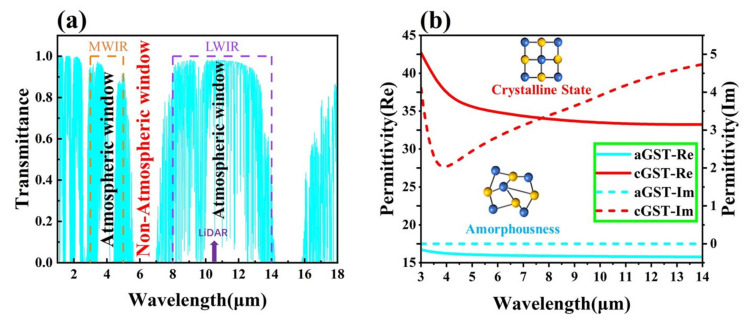
(**a**) Atmospheric transmission spectrum in the infrared band, schematic diagram of the LiDAR absorption band and the band where the atmospheric window is located. (**b**) Dielectric constants of crystalline GST and amorphous GST in the infrared band.

Since the photon energy in the infrared band is not much different from the chemical bond energy of various substances in the air, the atmosphere has a strong absorption effect on the light in the infrared band. Figure 2a shows the atmospheric transmission spectrum in the infrared band. Due to the high transmittance of the atmosphere in the mid-wave infrared (MWIR) and long-wave infrared (LWIR), light in the 3–5 μm and 8–14 μm bands can pass through atmosphere; these are called atmospheric windows. In the atmospheric window, the energy of an object can be directly radiated into the distance. The higher the radiation intensity, the easier it is for the infrared detector to absorb the energy of the corresponding band, and the easier it is to find the target. This is the principle of infrared detection. According to Kirchhoff’s law, under the condition of thermal equilibrium, we have

(3) α=ε, 

where α is the infrared absorption rate of the surface of the object and ε is the infrared radiation rate of the object. If one wants to avoid being detected by infrared detectors, the structure must have a low absorption rate/radiance rate (high reflectivity) in the atmospheric window to achieve the effect of infrared camouflage. Different from the principle of infrared camouflage, the physical mechanism of laser stealth is to achieve high absorption on the target surface. The working band of the laser is generally 10.6 μm. The light detection and ranging (LiDAR) detector emit laser light to the surface of the target, and then receives the reflected signals to detect the target. For LiDAR detection, we need to increase the absorptivity (reduce the reflectivity) of the target surface to achieve LiDAR stealth. As can be seen in Figure 2a, 10.6 μm is just in the atmospheric window band to achieve infrared camouflage. To achieve infrared-LiDAR compatible camouflage, it is necessary to ensure that the target has a high absorption rate at 10.6 μm and low absorption at 8–14 μm (except 10.6 μm). Figure 2b exhibits the dielectric constants of aGST and cGST in the infrared band. From the curves in the figure, we can observe that since the imaginary part of the dielectric constant (short blue line) is close to zero, aGST is a transparent medium in the infrared band without electromagnetic losses. When the GST phase changes to the crystalline state, the real part of the dielectric constant (solid blue and red lines) increases significantly, and the imaginary part also changes from zero. This phenomenon causes GST to generate electromagnetic losses in the infrared band and transform into an infrared absorbing material. In our study, selective infrared absorption of the device was first achieved through metasurface and nanogap resonance. Then, the nonvolatile PCM GST is used to perform dynamic infrared thermal radiation regulation and realize multifunctional applications.

### 3.1. “Wide-Angle, Polarization-Insensitive Dual-Band Infrared Detection” under the Four-Layer Structure

The first application of the designed multifunctional device is wide-angle, polarization-insensitive dual-band infrared detection. Infrared detection refers to finding the target by absorbing the radiation power of the target object in the mid-infrared band. Current infrared detectors can be roughly divided into two categories according to physical effects: the photothermal category and photonic category. They work in different ways, but the core principle is to determine the intensity of the target’s infrared radiation/absorption through changes in physical signals (such as geometry, conductivity) or electrical signals. Here, we only consider the radiation intensity of the target itself, while ignoring the radiation intensity of the surrounding environment and the solar radiation energy reflected by the target. The spectral radiation intensity of the black body and the target object can be expressed by Planck’s law of black body (BB) radiation, as follows [66]:

(4) $$I_{0}\left(\theta ,\lambda ,T\right)=\varepsilon \left(\theta ,\lambda ,T\right)I_{BB}\lambda ,T,\;$$

where $\varepsilon \left(\theta ,\lambda ,T\right)$ is the intrinsic emissivity of the object and $I_{BB}\lambda ,T$ represents the *BB* radiation spectrum. Simultaneously,

(5) $$I_{BB}(\lambda ,T)=\frac{2\pi hc^{2}}{\lambda^{5}}\cdot \frac{1}{e^{\frac{hc}{\lambda \kappa T}}-1}\;,\;$$

**Figure 3:**
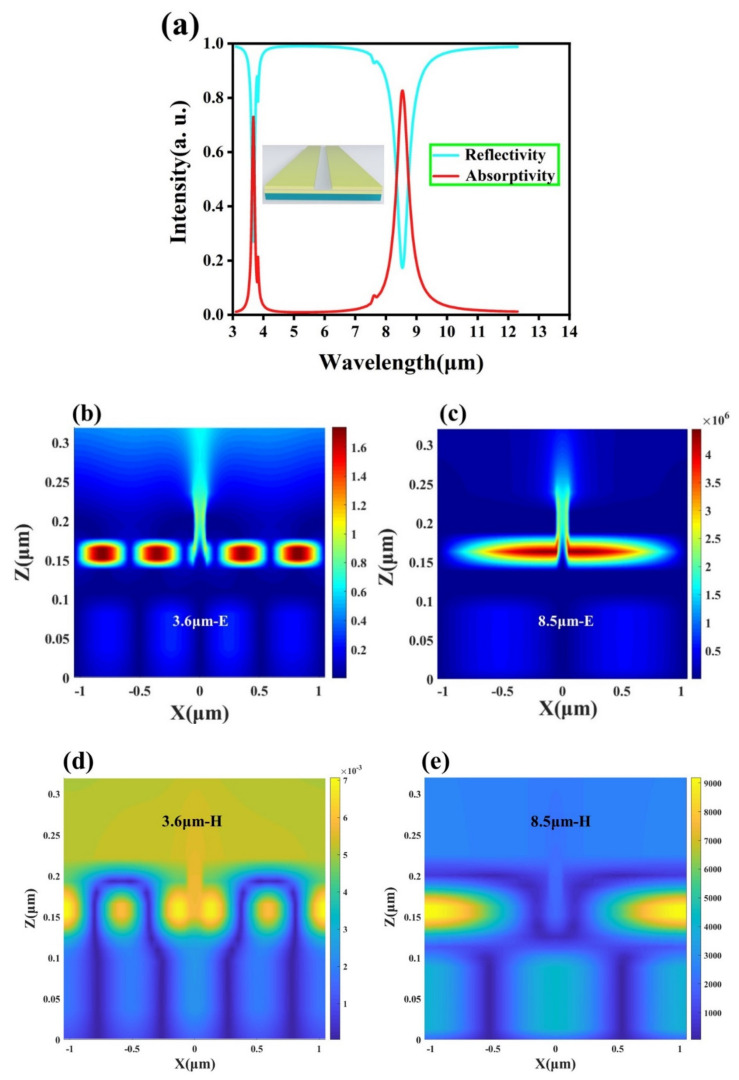
(**a**) Absorption and reflection spectra of the proposed four-layer structure in the infrared band. (**b**) Electric and (**d**) magnetic distributions at the first absorption peak. (**c**) Electric and (**e**) magnetic distributions at the second absorption peak.

This shows that $I_{BB}$ is only related to the temperature *T*, and all objects with a temperature higher than absolute zero can generate thermal radiation, such as the sun (6000 K), incandescent lamps (3000 K) or the human body (310 K) spontaneously radiating heat outward. Using this feature, the reconnaissance of target objects can be achieved by designing a device with a high absorption rate of thermal radiation at the atmospheric window. As shown in Figure 3a, the designed four-layer structure has obvious absorption peaks in the two atmospheric windows: the absorption rate reaches 73% at λ = 3.6 μm and the absorption rate is 83% at λ = 8.5 μm, respectively. It shows that this structure has a strong detection ability for infrared radiation of the target, and can be regarded as a mid-infrared detector. This is due to the introduction of narrow slits and nano-gap Al_2_O_3_ layers on the MIM structure. We found that they provide an important channel for controlling the absorption behavior; the former is to help the local energy leak out of the slit to be used away from the resonant unit, thus balancing the absorption of the material and the leakage rate of radiation and maintaining the perfect absorption of the structure, while the latter acts as a capacitor that can red-shift the absorption peak by adjusting the thickness and period [67]. In each constituent unit of Figure 1b, the dielectric spacing between the top gold array and the bottom metal can be viewed as a MIM waveguide. The round-trip between the ends of the metal array forms a waveguide-type FP cavity, where the lowest-order resonant mode results in high absorption at the corresponding wavelength. The absorption of this structure can be understood through time-domain coupled mode theory, which treats resonances as discrete modes that are weakly coupled to the environment. This device can be regarded as a typical gapped surface plasmon (*GSP*) resonator, and its resonance position can be described by a simple FP resonator equation [68,69]:

(6) $$w\frac{2\pi }{\lambda_{0}}n_{GSP}=m\pi -\phi ,\;$$

where $\lambda_{0}$ is the wavelength in free space, *w* is the band width, $n_{GSP}$ is the real part of the effective refractive index of the *GSP*, m is the mode order of the *GSP* and ϕ is an additional phase shift. It is worth pointing out that the *GSP* resonator is primarily an absorbing element when the gap is at subwavelength dimensions. Once the gap size becomes larger, the *GSP* resonance will gradually become weak enough to achieve high absorption. In order to explore the generation mechanism of the two absorption peaks located in the atmospheric window, we analyzed the electric and magnetic field distributions of the designed structure at λ = 3.6 μm and λ = 8.5 μm. Figure 3b,d show the electromagnetic field distribution at λ = 3.6 μm; we can find that the device excites high-order resonances at short wavelengths, and most of the electric field energy is trapped in the dielectric Al_2_O_3_ layer, indicating that nanogap resonances are generated and GSPs lead to absorption peaks. From Figure 3c, it can be seen that at λ = 8.5 μm, there is a clear coupling between the top gold array and the bottom metal, and opposite charges are observed at the corresponding positions, indicating the generation of localized surface plasmon resonance (LSPR). Combined with the magnetic field distribution in Figure 3e, it is found that the magnetic field energy is mainly concentrated in the gold array and the Au layer, indicating that there is a strong magnetic resonance (MR) in the dielectric region along the magnetic field direction. We believe that the emergence of MR can enhance the localized field at the resonance wavelength, thereby achieving high absorption at the corresponding wavelength through the enhanced LSPR. Therefore, we can use this phenomenon to realize dual-band infrared detection in the atmospheric window. Subsequently, we varied the thickness of the dielectric layer used to excite the nanogap resonance and found a significant change in absorptivity. This part of the work is detailed in Supplementary Materials Information S1. At the same time, we also verified that when the slit width is unchanged and the period becomes larger, the absorption peak has a red shift. It can extend this local resonance phenomenon to other wavelength bands, and it is fascinating for some applications that require subwavelength metasurface absorbing structures or strong interactions between light and matter.

**Figure 4:**
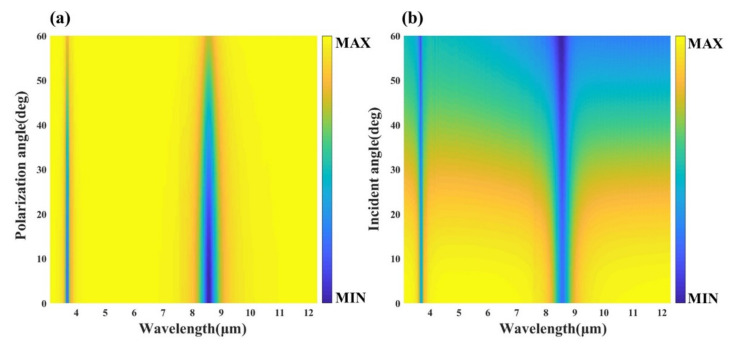
Reflectance spectra of the proposed four-layer structure in the infrared band (**a**) at different polarization angles and (**b**) at different incident angles.

Figure 4a shows the absorptivity of the device at different incident angles, and the absorptivity does not decrease significantly with the increase of the incident angle. Up to the large incident angle of about 60 degrees, the device can still maintain the absorption rate of more than 50% in the dual-band, indicating that it has good wide-angle absorption. Furthermore, since the designed structures are highly symmetric gold arrays and multilayer thin film structures, they are theoretically insensitive to polarization angles. Through the simulation calculation, we can know the influence of the polarization angle on the structure from Figure 4b, which verifies the polarization insensitivity of the device. Consequently, the designed four-layer structure exhibits wide-angle absorption and polarization insensitivity, which is beneficial for practical applications in infrared detection.

### 3.2. “Multifunctional Infrared Stealth, Radiation Cooling and Laser Stealth” under the Five-Layer Structure

The means of opposing infrared detection is infrared stealth technology, which is a matchup of “spear and shield”. Infrared stealth is to reduce the probability of the target being detected. The purpose is to make the radiation intensity of the target in the mid-infrared band consistent with the radiation intensity of the background, so that the detection imaging technology cannot distinguish between the two. In the mid-infrared band, because the radiation intensity of most of the background is very low, it is a key factor for infrared stealth to reduce the external radiation rate of the target through technical means. At the same time, detection systems on the battlefield no longer work only in the infrared band: multi-band detection is gradually becoming the mainstream thinking. With the continuous development of LiDAR technology, weapon systems using laser detection and guidance technology have high precision. Once the target is tracked by relevant weapons, the probability of survival will be extremely small. Therefore, the development of laser camouflage technology has great strategic significance. Laser camouflage technology is mainly aimed at 10.6 μm and other laser working bands. The purpose is to reduce the reflectivity of the target in these bands, so that the laser detection and guidance system cannot receive the reflected echo or identify the target. According to Kirchhoff’s law, the emissivity is equal to the absorptivity. At the same time, in traditional devices, it can be known from Equation (2) that for opaque materials, the sum of absorptivity and reflectivity is 1. Thus, to achieve compatible stealth in the IR band (3–5 and 8–14 μm) and the LiDAR band (10.6 μm), there is a natural conflict: the target needs to achieve both low absorbance/emissivity at 8–14 μm to achieve IR stealth and high absorbance (low reflectance) at 10.6 μm (in the 8–14 μm) to achieve LiDAR stealth. Therefore, it is still a very challenging task to achieve compatible camouflage in the IR-laser band.

**Figure 5:**
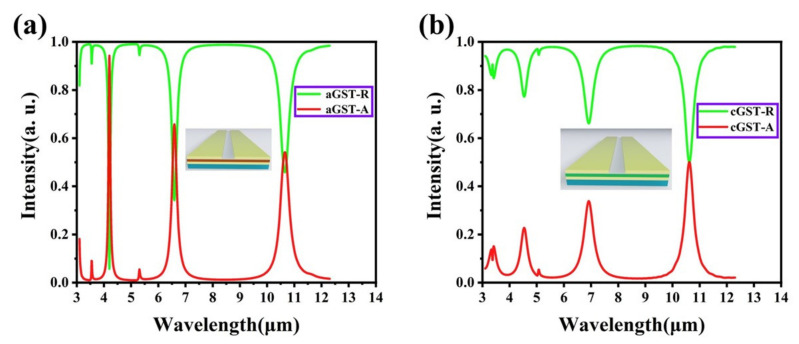
Absorptivity and reflectivity of the designed five-layer structure in the infrared-laser band (**a**) when the GST film is in the amorphous state and (**b**) when the GST film is in the crystalline state.

In response to the above practical requirements, we have researched and designed a multifunctional device, as shown in Figure 1d, which has a simple structure that can integrate multiple functions in a fixed structure. On the basis of the four-layer structure, we introduce the nonvolatile PCM GST, which can dynamically adjust the absorptivity of the device by using the change of its dielectric constant before and after the phase change, thereby realizing multifunctional applications. It can be observed from Figure 5 that after adding the GST film, the position and number of the main absorption peaks of the device have changed. This is because the resonance frequency and loss have changed, and the position and intensity of the resonance wavelength have also changed. Figure 5a shows the absorbance and reflectance of the designed five-layer structure in the IR-laser band. There is a clear absorption peak in each of the two atmospheric windows (3–5 and 8–14 μm) and in the non-atmospheric window (5–8 μm). Among them, the two absorption peaks located in the atmospheric window can reach the absorption rate of 95% and 55%, which still provides the basis for infrared detection. It is worth noting that the absorption/radiation intensity of the device in the non-atmospheric window can reach about 66%. At the same time, according to Boltzmann’s law [70],

(7) Eb=∫0∞Ebλdλ=∫0∞c1λ−5ec2λT−1dλ=σT4, 

where the proportionality coefficient σ is the Stefan constant, which is $\frac{2\pi^{5}k^{4}}{15c^{2}h^{3}}\approx 5.67\times 10^{-8}W\cdot m^{12}\cdot K^{-4},$ and it can be clearly found that the thermal radiation intensity of a black body is proportional to the fourth power of the temperature. The higher the temperature of the object, the greater the total energy radiated. Thus, we can control the radiation spectrum of objects by adjusting their intrinsic emissivity and their temperature. As shown in Figure 5, if we adjust the absorption/radiation wavelength to the non-atmospheric window by selective control, we can achieve effective thermal management by a higher absorption/radiation rate, so that heat can be released from the non-atmospheric window and heat buildup can be avoided. In this way, the device can achieve the effect of radiation cooling without being detected by the infrared detector. Based on the above phenomena, the first function of the five-layer structure we designed is infrared detection and radiation cooling. Then, simply by changing the temperature, after annealing the GST film at 160 °C (433.15 K), we obtained the five-layer structure containing the cGST film (thumbnail in Figure 5b). It can be observed from Figure 5b that the absorption rate of the device in the atmospheric window is significantly reduced, especially at 3–5 μm, where the maximum absorption rate is significantly reduced from 95% to 22%, and the average absorption rate is also reduced to about 15%. This makes it possible to transform functions of the device from infrared detection to infrared stealth.

**Figure 6:**
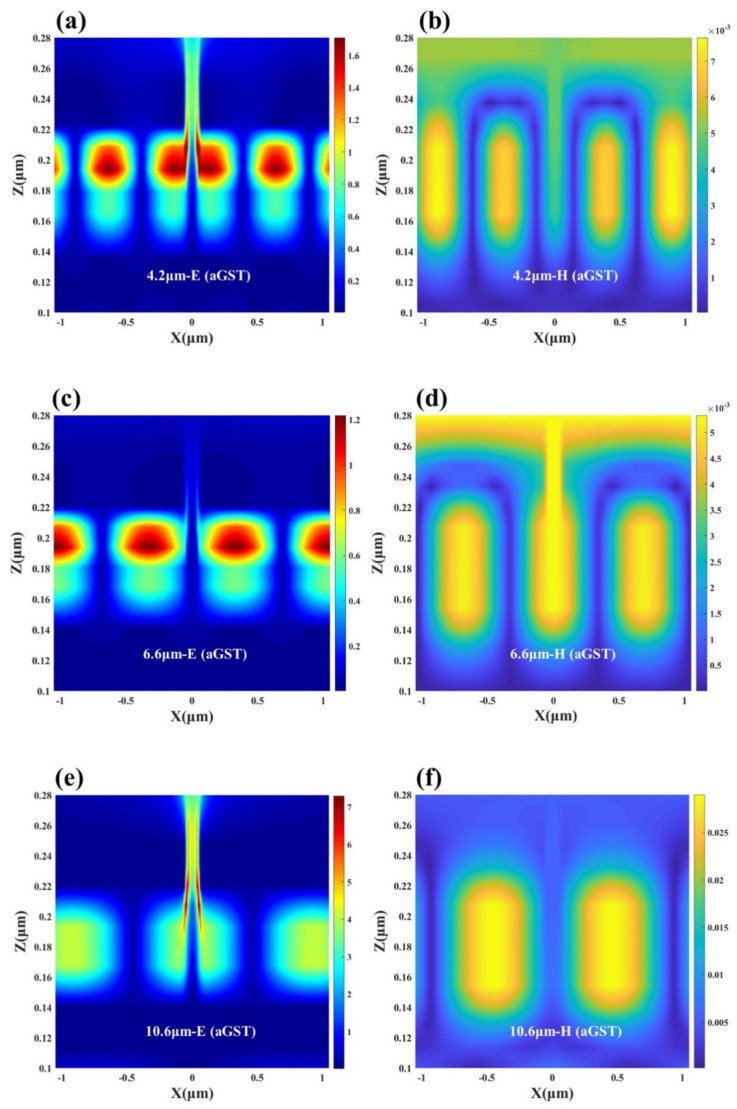
Schematic diagram of the electric and magnetic field distributions of the designed five-layer structure containing aGST thin films (**a**,**b**) at the first absorption peak, (**c**,**d**) at the second absorption peak and (**e**,**f**) at the third absorption peak.

**Figure 7:**
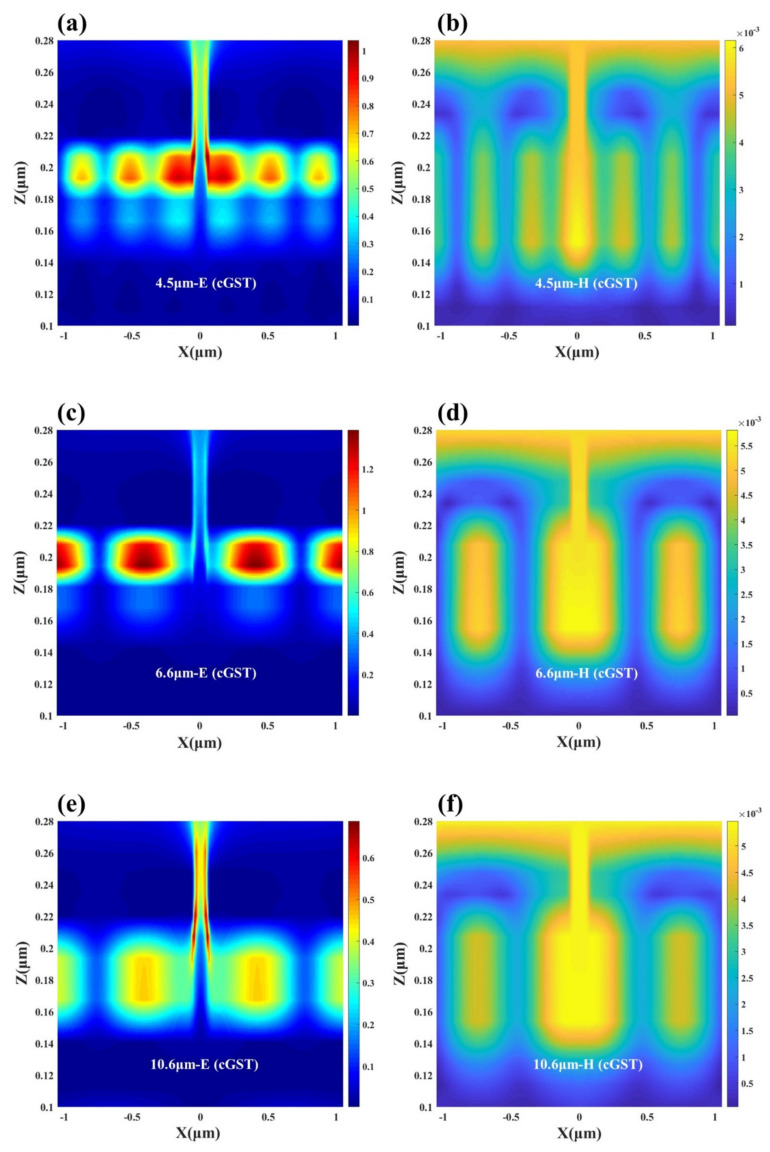
Schematic diagram of the electric and magnetic field distributions of the designed five-layer structure containing cGST thin films (**a**,**b**) at the first absorption peak, (**c**,**d**) at the second absorption peak and (**e**,**f**) at the third absorption peak.

In order to explore the reason for this phenomenon, we simulated and analyzed the electromagnetic field distribution of the device in two phase states. In the comparison of Figure 6a,b and Figure 7a,b, it can be found that the electromagnetic field intensity of the device at the first absorption peak decreases with the phase transition of GST. When the GST film is in an amorphous state, plasmon resonance occurs at the interface between the top surface of the underlying metal and GST, and the same resonance phenomenon is also excited at the interface between the top gold array and the nano-spaced Al_2_O_3_ layer. In addition, since the thickness of the dielectric layer is changed from 20 nm in the four-layer structure to a narrower 10 nm, a stronger nanogap resonance is excited, and the resonance wavelength is slightly red-shifted and the absorption effect becomes more intense. When the GST film phase changes to a crystalline state, various resonance effects are weakened, which leads to the transition of the device from high infrared absorption to low absorption. Similarly, by comparing Figure 6c,d with Figure 7c,d, we can observe that the electric field energy in the GST layer is significantly weakened, so that the absorption rate in the non-atmospheric window appears decreased: the absorption peak at λ = 6.6 μm decreased from 66% to 34%. However, since the magnetic field distribution is mainly concentrated at the slit and the connection area between the inner surface of the gold array and the thick gold layer, the change of the magnetic field strength is not large, and the effect of MR on the absorption spectrum is small. Although the average emissivity of the device in the non-atmospheric window has decreased at this time, it still maintains a certain radiative cooling effect. Interestingly, although the first two absorption peaks changed significantly, the absorption peak located at LWIR is exactly at *λ* = 10.6 μm, which is in a higher absorption state both before and after the phase transition. The position of the resonance wavelength is just the detection wavelength of LiDAR, so that better laser stealth function can be achieved. Combining Figure 6 and Figure 7 as a whole, it seems that both in the aGST and cGST states, higher order resonances are excited at short wavelengths, and the resonance order gradually decreases as the wavelength increases, which is also consistent with the properties of nano-gap resonance. Because gap plasmon resonance creates electric dipoles between parallel metal plates, smaller gap sizes result in lower energy states [71,72]. To sum up, it can be seen from Figure 5b that the average absorptivity/emissivity of the device in both atmospheric windows is around 20%. Especially under the premise of maintaining the high absorption in the 10.6 μm band, the average emissivity in the 8–14 μm band remains low. It shows that the five-layer structure can allow for the better infrared-laser stealth function, and at the same time has a certain radiation cooling effect, realizing multifunctional applications.

**Figure 8:**
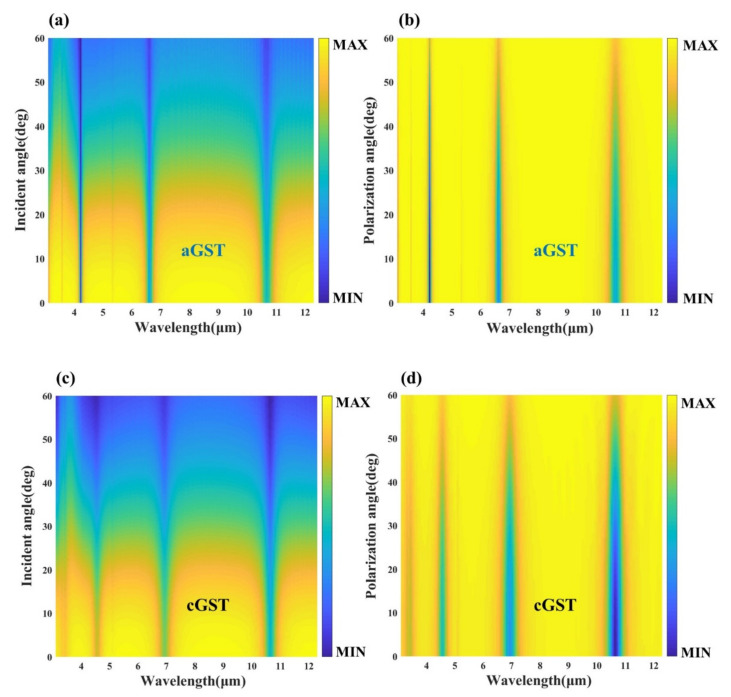
Reflectance spectra of the proposed five-layer structure in the infrared band: (**a**) aGST at different incident angles, (**b**) aGST at different polarization angles, (**c**) cGST at different incident angles and (**d**) cGST at different polarization angles.

Furthermore, the stability of the device under different incident and polarization angles is investigated. As shown in Figure 8, the reflectance spectra of the device under various conditions were obtained through simulation calculations. Regardless of whether the GST film is in the crystalline or amorphous state, the designed five-layer structure has good wide-angle absorption, and can still maintain high absorption at a large incident angle of about 60°. Meanwhile, the device also has good polarization insensitivity, and can maintain multifunctional applications well at a polarization angle of about 50°. This provides robust conditions for practical applications.

### 3.3. “Tunble Infrared-LiDAR Compatible Camouflage” by Different Crystalline Fractions of GST

It can be found in previous studies [63] that the main reason why the device can achieve multifunctional applications under the five-layer structure is the addition of GST [73]. In fact, the phase transition process of GST is continuous and reversible. The aGST and cGST can achieve mutual conversion under the stimulation of external temperature changes, electric field changes and direct laser irradiation. Since GST is a nucleation-oriented material, the amorphous GST is first obtained by magnetron sputtering. Then, the aGST will gradually form many small nuclei inside under the stimulation of external factors. Finally, these small nuclei will join together to form a regular crystal structure and become the cGST [74,75]. Therefore, it is theoretically possible to estimate the proportion of crystalline molecules in the GST films by the corresponding spectral simulations to derive the degree of GST phase transition. We assume that the GST films in the mesophase state consist of different proportions of amorphous and crystalline molecules with dielectric constants $\varepsilon_{aGST}\lambda$ and $\varepsilon_{cGST}\lambda$ at wavelength *λ*, respectively [76]. At the same time, we can apply the relevant effective dielectric theory to estimate the effective dielectric constant $\varepsilon_{GST}\lambda ,C$ of this GST film in the mesophase state, where C is 0% to 100% of this GST film (from aGST to cGST) crystalline fraction ratio. In this paper, we use Equation (8) to define the Lorentz–Lorentz relation to approximate the effective permittivity of the GST intermediate state [73]:

(8) $$\frac{\varepsilon_{GST}\lambda ,C-1}{\varepsilon_{GST}\lambda ,C+2}=C\times \frac{\varepsilon_{cGST}\lambda -1}{\varepsilon_{cGST}\lambda +2}+\left(1-C\right)\times \frac{\varepsilon_{aGST}\lambda -1}{\varepsilon_{aGST}\lambda +2},\;$$

**Figure 9:**
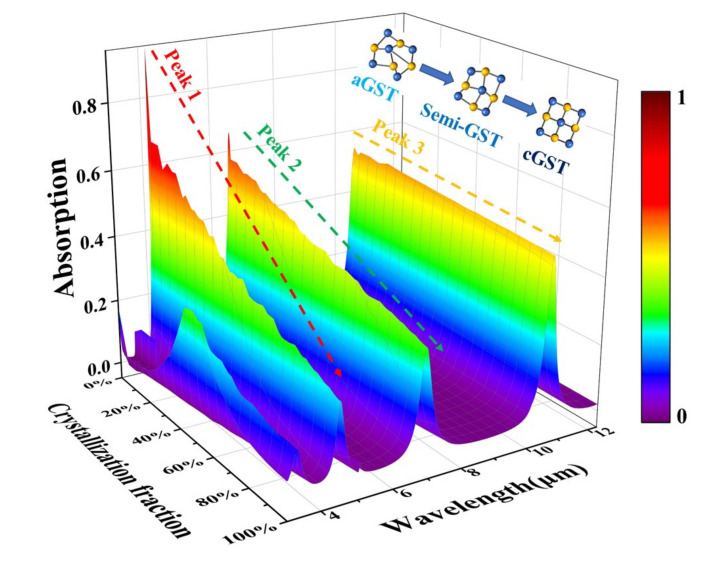
Three-dimensional schematic diagram of the absorbance of the designed five-layer structure as a function of the crystalline fraction of the GST film in the infrared band.

Thus, we can use the simple means of changing the temperature to stimulate the GST to be in different intermediate states. Further, the absorptivity of the device containing GST films with different crystalline fractions can be approximately calculated according to Equation (8). After simulation, we obtained a three-dimensional schematic diagram of the absorption rate of the designed five-layer structure in the infrared band, as shown in Figure 9. Figure 9 clearly shows the continuous changes of the three absorption peaks under each crystalline fraction of GST. It can be directly observed from Table 1 that there is a significant decrease in the absorptivity of the first absorption peak from aGST to cGST, and a weakening of the second absorption peak, while the third absorption peak changes very little.

Consequently, according to the dynamic change of the absorption rate of the device in different states of GST, we can use the significantly decreased absorbance of the first absorption peak to realize the functional transformation from IR detection to IR stealth in atmospheric windows. Good radiative cooling is maintained with good absorption/emissivity of the second absorption peak. The stable laser stealth function is realized by utilizing the almost invariant property of the third absorption peak. In addition, according to the continuous change of the GST phase transition process, we can achieve the corresponding IR-LiDAR compatible camouflage according to the actual emissivity of the background. The infrared emissivity of the device is made to be dynamically consistent with the background, so as to achieve dynamic and continuous infrared stealth and stable laser stealth. At the same time, the entire dynamic adjustment process can be achieved only by a simple temperature change, and the device can also maintain a certain radiative cooling when the temperature changes, so that the internal heat will not accumulate all the time, which is more in line with the requirements of practical applications. In practice, our proposed optical design can first be used for passive detectors in the infrared band. The metasurface detector can absorb the infrared radiation energy from the atmospheric window, and when the target enters the detection range, it causes a change in the absorbed energy. By comparing with the ambient energy, passive infrared detection can be achieved. Furthermore, the proposed optical design can be mounted on the outer surface of the protected device to achieve active laser stealth and passive IR stealth. By the higher absorption at 10.6 μm, this design can absorb most of the active LiDAR detection signals and make the signals impossible to return. At the same time, the device can avoid detection by IR detectors due to its low emissivity in the atmospheric window when the design contains the cGST. Finally, the proposed optical design can also wrap the device inside and provide energy release and passive radiative cooling for the device using the emissivity in the non-atmospheric window. In conclusion, our device realizes complex multi-band, multi-scenario and multifunctional applications on a fixed and compact structure, providing important ideas and references for electromagnetic shielding, perfect absorption, thermal management and infrared stealth.

## 4. Conclusions

In summary, this paper proposes a multifunctional nanostructure with phase-change materials for tunable infrared detection, radiative cooling and infrared-laser compatible camouflage. The structure uses a combination of gold arrays with slits in the top-layer, and innovatively introduces the nonvolatile phase-change material GST. The study shows that by combining the plasmon resonance and nanogap resonance excited from the classical MIM structure, the device can selectively tune the absorptivity/emissivity at specific wavelengths. Due to the symmetry of the structure, the proposed device has the remarkable features of wide-angle absorption and polarization insensitivity, which is beneficial for practical applications. At the same time, according to the analysis of the energy distribution of the electromagnetic field and the study of various resonance theories, we can expand the strong interaction between light and matter to other wavelengths and achieve more applications. It should be emphasized that, different from the single function of other traditional structures, the proposed multifunctional structure can realize complex multiple functions without changing the structure by only changing the temperature. In addition, our device is extremely simple and easy to fabricate, which will greatly benefit the needs of integration and miniaturization. It has potential applications in various fields such as electromagnetic absorbers, electromagnetic shielding, infrared stealth, optical communication and sensors.

## Figures and Tables

**Table 1:** Absorptivity variation of the main absorption peaks of the device at different crystalline fractions of GST.

Crystallization Fraction /Wavelength	0% (aGST)	25%	50%	75%	100% (cGST)
Peak1 (4.2 μm–4.5 μm)	95%	57.7%	42.5%	31.9%	22%
Peak2 (6.6 μm)	66%	54%	45.6%	38.8%	34%
Peak3 (10.6 μm)	55%	53.9%	52.4%	51.3%	51%

## Data Availability

Data are available in the main text.

## Supplementary Materials

The following supporting information can be downloaded at: https://www.mdpi.com/article/10.3390/nano12132261/s1, Figure S1: Effect of structural parameters of the proposed nanostructure on the absorptivity of the four-layer structure: (a) variation of the thickness of the nanogap Al_2_O_3_ layer, and (c) variation of slit width, Figure S2: Effect of structural parameters of the proposed nanostructure on the absorptivity of the five-layer structure: (a) variation of the thickness of the nanogap Al_2_O_3_ layer, (b) variation of the thickness of the GST layer and (c) variation of slit width, Figure S3: Fabrication schematic of the proposed multifunctional device: (a) deposition of 100 nm thick Si layers via a magnetron sputtering tool; (b–e) successive addition of the Au film (50 nm), the GST film (50 nm), the Al_2_O_3_ film (10 nm) and the Au top-layer film (50 nm) on the top of the underlying structure by magnetron sputtering; (f) spin-coating of a thick photoresist layer on the Au layers; (g) fabrication of the photoresist top-layer structure by photolithograph; (h) etching to transfer the top-layer structure from the photoresist layer to the Au layer; and (i) cleaning and removal of the residual photoresist arrays.
