# Supplementary material for: Tunable Infrared Detection, Radiative Cooling and Infrared-Laser Compatible Camouflage Based on a Multifunctional Nanostructure with Phase-Change Material
Source: Nanomaterials (Basel). 2022 Jun 30;12(13):2261. doi: 10.3390/nano12132261 (PMC9268176; doi:10.3390/nano12132261)
Supplement: Supplementary file 1 [file nanomaterials-12-02261-s001.zip › nanomaterials-1728923-supplementary.pdf]

Supplementary Material

Tunable Infrared Detection, Radiative Cooling and Infrared-Laser Compatible Camouflage Based on a Multifunctional Nanostructure with Phase-Change Material

Mingyu Luo [1,2,†], Xin Li [2,†], Zhaojian Zhang [2], Hansi Ma [2], Te Du [2], Xinpeng Jiang [2], Zhenrong Zhang [1,*] and Junbo Yang [2,*]

[1] Guangxi Key Laboratory of Multimedia Communications and Network Technology, School of Computer, Electronics and Information, Guangxi University, Nanning 530004, China; luomingyu20@163.com

[2] Center of Material Science, College of Liberal Arts and Sciences, National University of Defense Technology, Changsha 410073, China; lixin1010106@163.com (X.L.); zhangzhaojian@nudt.edu.cn (Z.Z.); mahansi21@nudt.edu.cn (H.M.); dute95@foxmail.com (T.D.); jackson97666@163.com (X.J.)

[†] These authors contributed equally to this work.

[*] Correspondence: zzr76@gxu.edu.cn (Z.Z.); yangjunbo@nudt.edu.cn (J.Y.)

Supplementary Materials Information S1. Effect of structural parameters of the proposed multi-functional nanostructure on the absorptivity

In order to study and design such nanostructures more comprehensively, we make a further discussion on the effect of its structural parameters on the absorptivity of nanostructure. The absorption spectra of the proposed device at different slit width and layer thickness are shown in the Figures S1 and S2.

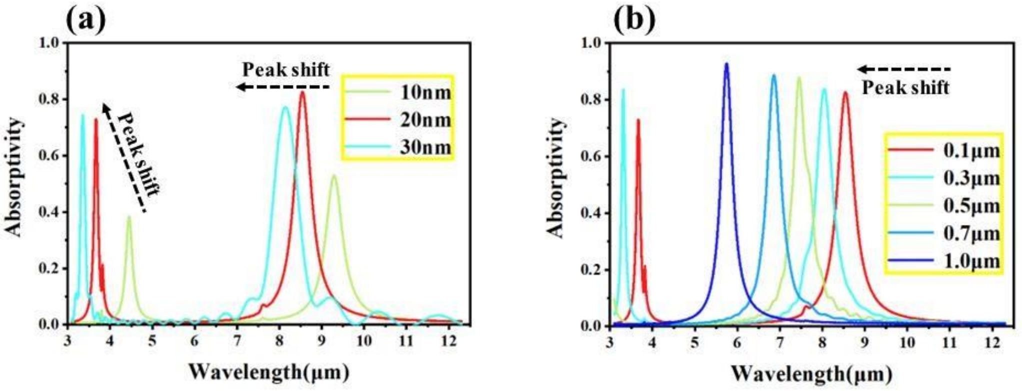

Figure S1. Effect of structural parameters of the proposed nanostructure on the absorptivity at the four-layer structure. (a) Variation of the thickness of the nanogap Al_2O_3 layer. (b) Variation of slit width.

The simulation results show that when the device is in a four-layer structure, the variation of the thickness of the nanogap Al_2O_3 layer has a significant effect on the absorptivity, while the variation of the thickness of the Au layer has little effect. This is because the nanogap resonance occurs mainly in the Al_2O_3 layer, and the strength of the resonance leads to a significant change in the absorptivity. So, we analyzed the results brought by the variation of the thickness of the Al_2O_3 layer. From Figure S1a, it can be seen that when the thickness of Al_2O_3 layer changes from 10 nm to 30 nm, both absorption peaks will be blue-shifted and the maximum value of the absorptivity will also change. In addition, Figure S1b shows the effect of the width of the slit in the top layer on the absorptivity. We can clearly find that if the period is constant, the absorption peaks gradually blueshift

as the slit becomes wider and wider. At the width of 0.5 μm, only one absorption peak is left in the absorption spectrum. It is due to the localized surface plasmon resonance (LSPR) and magnetic resonance (MR) mainly concentrated in the top Au array, changing the slit width ofthe Au array will directly affect the resonance intensity and thus cause the change of absorptivity.

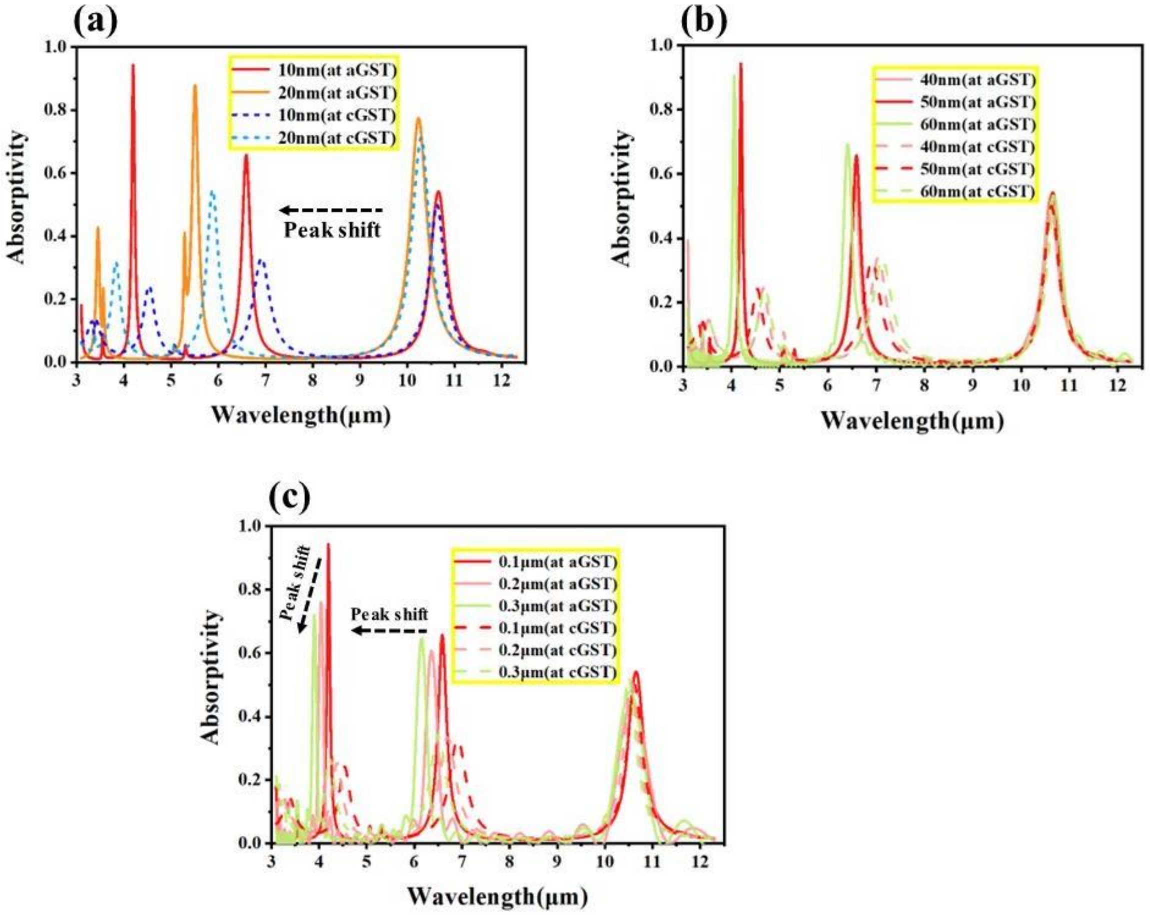

Figure S2. Effect of structural parameters of the proposed nanostructure on the absorptivity at the five-layer structure.(**a**) Variation of the thickness of the nanogap Al_2O_3 layer. (**b**) Variation of the thickness of the GST layer. (**c**) Variation of slit width.

Similarly, the variation of the thickness of the nanogap Al_2O_3 layer still has a significant effect on the absorptivity at the five-layer structure. As shown in Figure S2a, the increase of thenanogap layer thickness leads to the blue-shift of the absorption peak both at aGST and at cGST. We find that the best LiDAR stealth can be achieved only at a thickness of 10 nm, whenthe absorption peak of LWIR is just at 10.6 μm. At this point, the strong absorption from the resonance allows the device to achieve multifunctional applications. In addition, we discuss the results brought by the thickness variation of the newly added GST layer. In Figure S2b, it can be clearly found that the GST thickness changes from 40 nm to 60 nm, but the absorptivity changes very little. This is consistent with our theoretical analysis, because the intensity of the main resonance generated is not determined by the GST layer. Instead, the main role of the GST is to use the difference of the dielectric constant to make the resonant frequency and loss change before and after the phase transition, thus affecting the resonant wavelength. Finally, we similarly discuss the effect of the width of the slit in the top layer structure on the absorptivity. From the results shown in Figure S2c, we can know that the best result of infrared detection and radiative cooling function can be achieved when we choose a slit width of 0.1 μm.

Supplementary Materials Information S2 Description of fabrication steps

Due to the wide application of nanostructure design [1,2], electron beam lithography (EBL) has attracted more and more attention, and it may be used to fabricate spectral sensors [3], matefilms [4] and various nanostructures [5–7]. Zhu et al. fabricated the super-surface withdouble layer structure by utilizing resonant laser printing of structural colors, and its period size is 200nm [8]. And Zhang et al. design one-dimensional doping-structure photonic crystals to realize compatible stealth, which contain twelve film layers and complex doping [9]. Compare with the above nanostructure fabrication, the proposed design has larger periodsizes, simpler top-level structure and fewer film layers, which will be more feasible to fabrication. We refer to the lithography and reactive ion etching (RIE) processing, as well as the deposition technology of film layers and experimental fabrication of optical devices [10]. To make the process clearer, we give the concise processing steps, as shown in Figure S3.

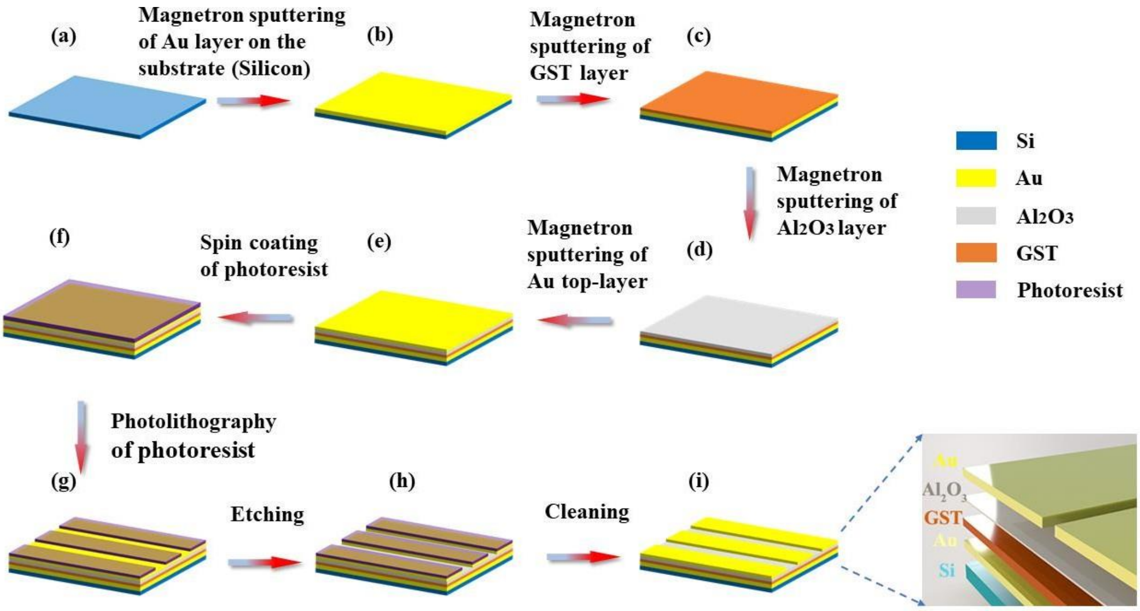

Figure S3. Fabrication schematic of the proposed multi-functional device. (**a**) Deposition of 100 nm thick Si layers via a magnetron sputtering tool. (**b–e**) the Au film (50 nm), the GST film (50 nm), the Al_2O_3 film (10 nm) and the Au top-layer film (50 nm) are successively added on the top of the underlying structure by magnetron sputtering. (**f**) Spin-coating of a thick photoresist layer on the Au layers. (**g**) Fabrication of the photoresist top-layer structure by photolithograph. (h) Etching to transfer the top-layer structure from the photoresist layer to the Au layer. (**i**) Cleaningand removing the residual photoresist arrays.

The proposed multi-functional device is fabricated in four steps: First, the Si layers with a thickness of 100 nm is deposited on the substrate via a magnetron sputtering tool in Figure S3a. Then, as shown in Figure S3b–e, the Au film, the GST film, the Al_2O_3 film and the Au top-layer film are successively added on the top of the underlying structure by magnetron sputtering. Besides, Figure S3f–h illustrate fabrication of the photoresist layers and etching to transfer the top-layer structure from the photoresist layer to the Au layer. Finally, an acetone solution is used to remove and clean the residual photoresist fractal structure as shown in Figure S3i.

References

1. Yoon, G.; Kim, I.; So, S.; Mun, J.; Kim, M.; Rho, J. Fabrication of three-dimensional suspended, interlayered and hierarchical nanostructures by accuracy-improved electron beam lithographyoverlay. *Scientific reports* **2017**, *7*, 1–8.
2. Bang, S.; Kim, J.; Yoon, G.; Tanaka, T.; Rho, J. Recent advances in tunable and reconfigurablemetamaterials. *Micromachines* **2018**, *9*, 560.
3. Stewart, J.W.; Vella, J.H.; Li, W.; Fan, S.; Mikkelsen, M.H. Ultrafast pyroelectric photodetection with on-chip spectral filters. *Nat. Mater.* **2020**, *19*, 158–162.
4. Lee, T.I.; Cho, J.W.; Jeong, D.; Lee, K.J.; Park, B.Y.; Kim, T.J.; Kim, Y.D.; Kim, Y.S.; Nam, Y.; Kim, S.K. Pt/Alumina Hyperbolic Metafilms with High-Temperature Stability, Wide Wavelength Tunability, and Omnidirectional Absorption. *Phys. Status Solidi (A)* **2018**, *215*, 1800287.
5. Kubis, M.; Wise, R.; Chahine, C.; Viatkina, K.; Ur-Rehman, S.; Simons, G.; Dusa, M.; Hellin, D.; Sobieski, D.; Zhang, W. Reducing the impact of etch-induced pattern shift on overlay by using lithography and etch tool corrections. *Optical Microlithography XXX*, (SPIE, **2017**), 108–116.
6. Dyachenko, P.N.; Molesky, S.; Petrov, A.Y.; Störmer, M.; Krekeler, T.; Lang, S.; Ritter, M.; Jacob, Z.; Eich, M. Controlling thermal emission with refractory epsilon-near-zero metamaterials via topological transitions. *Nat. Commun.* **2016**, *7*, 1–8.
7. Zhai, Y.; Ma, Y.; David, S.N.; Zhao, D.; Lou, R.; Tan, G.; Yang, R.; Yin, X. Scalable-manufactured randomized glass-polymer hybrid metamaterial for daytime radiative cooling. *Science* **2017**, *355*, 1062–1066.
8. Zhu, X.; Yan, W.; Levy, U.; Mortensen, N.A.; Kristensen, A. Resonant laser printing of structuralcolors on high-index dielectric metasurfaces. *Science Advances* **2017**, *3*, e1602487.
9. Zhang, J.–K.; Shi, J.–M.; Zhao, D.–P.; Wang, Q.–C.; Wang, C.–M. Realization of compatible stealth material for infrared, laser and radar based on one-dimensional doping-structure photonic crystals.*Infrared Physics & Technology* **2017**, *85*, 62–65.
10. Wu, S.; Gu, Y.; Ye, Y.; Ye, H.; Chen, L. Omnidirectional broadband metasurface absorber operating invisible to near-infrared regime. *Optics Express* **2018**, *26*, 21479–21489.
